# Evidence for altered neurodevelopment and neurodegeneration in Wolfram syndrome using longitudinal morphometry

**DOI:** 10.1038/s41598-019-42447-9

**Published:** 2019-04-12

**Authors:** Heather M. Lugar, Jonathan M. Koller, Jerrel Rutlin, Sarah A. Eisenstein, Olga Neyman, Anagha Narayanan, Ling Chen, Joshua S. Shimony, Tamara Hershey

**Affiliations:** 1 Department of Psychiatry, Washington University School of Medicine, St. Louis, Missouri USA; 2 Mallinckrodt Institute of Radiology, Washington University School of Medicine, St. Louis, Missouri USA; 3 Department of Biostatistics, Washington University School of Medicine, St. Louis, Missouri USA

## Abstract

Wolfram syndrome is a rare disease caused by mutations in the *WFS1* gene leading to symptoms in early to mid-childhood. Brain structural abnormalities are present even in young children, but it is not known when these abnormalities arise. Such information is critical in determining optimal outcome measures for clinical trials and in understanding the aberrant neurobiological processes in Wolfram syndrome. Using voxel-wise and regional longitudinal analyses, we compared brain volumes in Wolfram patients (n = 29; ages 5–25 at baseline; mean follow-up = 3.6 years), to age and sex-equivalent controls (n = 52; ages 6–26 at baseline; mean follow-up = 2.0 years). Between groups, white and gray matter volumes were affected differentially during development. Controls had uniformly increasing volume in white matter, whereas the Wolfram group had stable (optic radiations) or decreasing (brainstem, ventral pons) white matter volumes. In gray matter, controls had stable (thalamus, cerebellar cortex) or decreasing volumes (cortex), whereas the Wolfram group had decreased volume in thalamus and cerebellar cortex. These patterns suggest that there may be early, stalled white matter development in Wolfram syndrome, with additional degenerative processes in both white and gray matter. Ideally, animal models could be used to identify the underlying mechanisms and develop specific interventions.

## Introduction

Wolfram syndrome (OMIM #222300) is a rare (1 in 500,000 to 1,000,000) autosomal recessive genetic disease originally described as the combination of insulin dependent diabetes mellitus, optic nerve atrophy, diabetes insipidus and deafness^1^. Neurodegeneration and neurological features were thought to appear at later stages of the disease, ultimately leading to death in middle adulthood^2^. Since the major causative gene (*WFS1*)^3^ was identified in 1998, greater variability in the severity of the disease, the complexity of the phenotype and the rate of progression has been revealed. It is now evident that not all of the presumed core symptoms of Wolfram syndrome are present in all of the patients who are genetically identified^4–6^ and that neurologic abnormality is present even at early stages of the disease in some patients^7–9^. Rare neuropathological examinations of Wolfram syndrome patients’ brains have noted loss of neurons, myelin and myelinated axons^10–14^; however it is unclear when in the disease process these occur. Given that the neurological features of Wolfram syndrome are life-threatening and evolve over development, these aspects should be targets of intervention and thus require greater attention.

Mutations in the *WFS1* gene predispose pancreatic β-cells to ER stress-mediated apoptosis^15–22^ and this mechanism may also account for central nervous system degeneration, including, perhaps, myelin degradation^23^. However, this idea has not been well tested in brain cells, nor do we understand how this mechanism interacts with neurodevelopmental processes. While molecular and cellular experiments have led the field closer to identifying potential interventions for degenerative processes in Wolfram syndrome, the identification of reliable neurological biomarkers and an understanding of underlying neuropathophysiology and clinical correlates is in its infancy. For these reasons, determining the trajectory of structural brain changes in Wolfram syndrome during development is a critical step forward for the field.

In our previous cross-sectional neuroimaging study, individuals with Wolfram syndrome, as compared to age and sex equivalent controls, had reduced gray and white matter volumes primarily in the ventral pons, brainstem, cerebellum, and thalamus with altered white matter microstructural integrity in tracts such as optic radiations^9,24^. Interestingly, decreased brainstem and cerebellar volumes were seen in almost all individuals with Wolfram syndrome, regardless of age or duration of diabetes or other features of the disease. These data suggested that Wolfram syndrome affects the early development of specific brain regions. However, cross-sectional comparisons cannot determine whether these abnormalities evolve over time within an individual or at what rate, nor how they are distinguished from normal developmental trajectories. Thus, we have performed yearly neuroimaging studies in patients with Wolfram syndrome and controls over an age range of active neurodevelopment. By examining morphometric change with multiple spatial and statistical methods, we aim to converge on the regions and tissue types that are most affected in Wolfram syndrome, distinguish between earlier vs. later occurring abnormalities and identify rates of regional change that diverge from normal neurodevelopmental trajectories. Findings may indicate future targets for brain-specific intervention and identify outcome measures for clinical trials targeting neurological symptoms. It will also greatly expand our understanding of the longitudinal phenotype of *WFS1*-related disorders, rather than classically defined Wolfram syndrome. Such knowledge will have a significant impact on patients and families by allowing physicians to provide more accurate prognoses.

## Methods

### Participants

This study was approved by the Human Research Protection Office at Washington University in St. Louis and all methods were conducted in accordance with relevant guidelines and regulations. Children under age 18 gave informed assent and parents/guardians gave informed, written consent. Participants 18 or older gave informed, written consent.

#### Wolfram syndrome group

Patients were recruited into the Washington University Wolfram Syndrome Research Clinic through self or physician referral or from the Washington University Wolfram Syndrome Registry. Patients had to have known *WFS1* mutations, be under the age of 30 at enrollment and aware of their diagnosis, and be able to travel to St. Louis for annual research clinic visits. Patients were evaluated annually by physician specialists, answered questionnaires and performed different tests. Data from some of these measures have been previously reported^5,7–9,25–34^. Each patient was also assessed by a neurologist who performed the Wolfram Unified Rating Scale (WURS), a scale designed to measure the severity of symptoms commonly associated with Wolfram syndrome^26^. We used the physical subscale as a measure of neurologic symptom severity. Wolfram patients without contraindications also underwent magnetic resonance imaging (MRI) at each visit.

#### Control group

Healthy individuals with and without type 1 diabetes mellitus composed the control group. Participants with type 1 diabetes were recruited from the Pediatric Diabetes Clinic at St. Louis Children’s Hospital and Washington University School of Medicine in St. Louis. They were excluded for diagnosed psychiatric disorder, significant neurological history not due to diabetes, known premature birth (<36 weeks gestation) with complications, psychoactive medications, or physical limitations that would interfere with testing. No participants had known retinopathy, nephropathy or neuropathy at the time of testing. Non-diabetic individuals were either healthy siblings of the participants with diabetes or from the community. Non-diabetic controls were excluded for known current or past history of neurological and psychiatric diagnoses or other significant health conditions. Each participant in these control groups underwent MRI scans, answered questionnaires and completed cognitive tests at each annual visit for up to 3 years.

### Neuroimaging

Before beginning scanning, participants with diabetes were determined to always have blood glucose levels above 70 mg/dl, and below 300 mg/dl when possible. Scanning was performed on a single Siemens 3T Tesla Tim Trio at Washington University and included T1 and T2-weighted sequences. The T1-weighted Magnetization-Prepared Rapid Gradient-Echo (MPRAGE) sequence had the following parameters: sagittal acquisition, repetition time (TR) = 2400 ms, echo time (TE) = 3.16 ms, inversion time (TI) = 1000 ms, voxel resolution = 1 × 1 × 1 mm, time = 8:09 min. The T2-weighted sequence had the following parameters: sagittal acquisition, TR = 3200 ms, TE = 455 ms, voxel resolution = 1 × 1 × 1 mm, time = 4:43 min. All imaging sessions with acceptable T1 and T2 scans were selected for analyses. Diffusion-weighted, limited field of view T2, and resting BOLD scans were also attempted, but data are not reported here.

Analysis methods were selected to provide complementary and convergent information on how gray and white matter volumes change over time in Wolfram syndrome and controls. We used a voxel-wise imaging analysis approach that disregarded anatomical boundaries and tissue types and a region of interest (ROI) method, independently analyzed with appropriate statistical models for varying numbers of time points across participants. Since previous analyses have not found differences between healthy and diabetic groups^9,24^, we pooled the control groups and included the presence of diabetes as a covariate in the model to simplify statistical analyses and to maximize power.

#### Voxel-wise analysis methods

For each participant with two or more time points, a T1 and a T2 from each time point was processed using the serial longitudinal registration toolbox in SPM 12 with default settings^35^. For each time point per participant, the T2 was co-registered to the corresponding T1 from that visit. The deformation maps from the T1 longitudinal registration were then used to sample the T1 and T2 for each time point onto the mid-point average T1 longitudinal registration space and make an all-time-point average for both the T1 and T2. The all-time-point average T1 and T2 were used as inputs for SPM segment^36^. Using both the T1 and T2 images in SPM segment can improve segmentation accuracy (https://www.fil.ion.ucl.ac.uk/spm/doc/manual.pdf). The resulting gray and white matter tissue probability maps (TPMs) for each participant were used to create study-specific gray and white matter templates using SPM’s DARTEL^37^. These templates were used to align each subject’s gray and white matter TPMs to a common group template space. To achieve a better correspondence with the MNI152 VI asymmetric template (MNI152_VIa) (http://nist.mni.mcgill.ca/?page_id=714), we segmented the MNI152_VIa T1 template image with SPM segment and used SPM DARTEL to compute a mapping between MNI152_VIa and the common group template created from our data. For each person, we then took their average gray and white matter TPMs and the Jacobian determinant maps for each time point and warped them into MNI152_VIa space using the composite warp from subject space to MNI152_Via space. Each Jacobian determinant map (tensor-based morphometry map, “TBM”) represents degree of expansion or contraction from a template (subject or group) at the voxel level. The group-average gray and white matter TPMs were used to create a mask for voxel-wise analyses.

The sandwich estimator toolbox (SwE, version 1.2.8 for SPM) was used to statistically analyze TBM’s^38^. The SwE toolbox optimizes the analysis of longitudinal neuroimaging data by accommodating datasets that are small, unbalanced, and have missing data. SwE applies a non-iterative, marginal model to the data which allows each group to have its own covariance matrix and prevents within-subject convergence problems inherent in longitudinal designs. The main effect of group (Wolfram vs controls) was tested by using the TBMs for time-point-to-group-template, while controlling for estimate of total intracranial volume (eTIV^39^), age, sex, and diabetes. Annualized change in volume (defined as time in years from each time point to the mid-point for each TBM) was modelled as a fixed effect. The interactions of group by time by age and of group by time, as well as the main effect of time within each group were tested using the TBMs for time-point-to-midpoint-average while controlling for age, sex, and diabetes. The wild bootstrap (WB) component of the SwE toolbox was used to make non-parametric inferences about cluster level data using family-wise error (FWE) correction (http://hdl.handle.net/2268/186284). A cluster-forming threshold of p = 0.001 was used for the 999 bootstrap permutations, and FWE-corrected cluster-level p values were considered significant at p < 0.05.

#### Regions of interest (ROI) methods

For all participants with a T1 from at least one time point, the semi-automatic segmentation program Freesurfer (v5.3) was used to extract global and regional gray and white matter volumes at each time point using the longitudinal processing stream^24,40^. Specific regional volumes were selected for analysis to match the location of major clusters on voxel-wise group by time interaction maps that survived FWE correction. Regional volumes were averaged across left and right hemispheres when appropriate and corrected for eTIV^39^. Volumes for ventral pons were measured by first aligning the Freesurfer brainstem ROIs for each participant across all time points and then averaging them. Manual segmentation was then performed on each individual’s average brainstem ROI using previously described methods^24^ before applying those segments back to each time point, and extracting eTIV-corrected volumes^9,24^.

Statistical analyses assumed a linear relationship between regional brain volumes and time and used a random slope model (mixed model) to predict average annual rate of change for each regional volume. This model allowed slopes (annual rate of change) to vary randomly between participants and fit a separate regression line for each participant. The three-way interaction between group, time and age at the first testing session and all the two-way interactions, as well as sex and diabetes effect, were considered in a full model. Backward selection method was used to remove the most non-significant factor, one at each step, in the order of three-way interaction, two-way interactions and main effects. Analyses were two-tailed with a significance level of 0.05 and performed with SAS 9.4 (SAS Institute, Cary, NC).

Power analyses were performed on the region with the greatest degree of abnormal change within just the Wolfram group to determine the sample size necessary to detect a significant effect of intervention in a clinical trial. Simulations were conducted to determine the sample size required to achieve at least 80% power to detect a 50% or 60% reduction in mean annual rate of progression at significance level of 0.05. Analyses assumed that measurements would occur every six months during a 3-year clinical trial, and that sample sizes and mean volumes at baseline were equal for the two Wolfram groups (treatment vs. placebo). Mean trajectories were simulated using the random slope model, using a mean annual rate of progression for the placebo group estimated from the current Wolfram data. Simulations assumed random slope and homoscedastic error normally distributed with zero mean and variance estimated from the Wolfram group data. Empirical power was calculated from 1000 simulated trials.

## Results

### Participants

#### Wolfram syndrome group

Adequate T1 and T2 images were acquired on 29 patients with Wolfram syndrome seen annually between 1 and 7 times (mean follow-up = 3.64 years) (Table 1).

**Table 1:** Mean and standard deviation (SD) of demographic and clinical variables across Wolfram and control groups at session 1.

Variable		Wolfram (n = 29)	Controls (n = 52)	Control Subsets	
				Non-diabetic controls (n = 28)	Diabetic controls (n = 24)
Sex	M/F	15/14	27/25	13/15	14/10
Age (years)	Mean ± SD	13.8 ± 5.5	13.9 ± 5.0	14.1 ± 5.3	13.6 ± 4.7
	Range	5.9–25.8	6.0–26.2	6.0–26.1	7.6–26.2
*Follow-up period (years)	Mean ± SD	3.6 ± 1.7	2.0 ± 0.3	1.9 ± 0.3	2.0 ± 0.2
	Range	1.0–5.9	1.0–2.3	1.0–2.3	1.1–2.3
Diabetes duration (years)	Mean ± SD	7.9 ± 5.2	—	—	7.8 ± 4.8
	Range	0.4–16.6	—	—	0.1–16.7
Diabetes mellitus	Prevalence	29/29	—	—	—
Age at dx (years)	Mean ± SD	5.9 ± 3.0	—	—	—
	Range	2.7–13.9	—	—	—
Diabetes insipidus	Prevalence	18/29	—	—	—
Age at dx (years)	Mean ± SD	11.0 ± 3.8	—	—	—
	Range	5.5–17.0	—	—	—
Hearing loss	Prevalence	20/29	—	—	—
Age at dx (years)	Mean ± SD	10.5 ± 4.7	—	—	—
	Range	6.0–25.8	—	—	—
Optic nerve atrophy	Prevalence	28/29	—	—	—
Age at dx (years)	Mean ± SD	10.1 ± 3.9	—	—	—
	Range	3.8–18.0	—	—	—

There were no differences between the Wolfram and control groups for age (p = 0.93) or sex (p = 0.99), and there were no differences between Wolfram and the diabetic control groups for duration of diabetes (p = 0.95) at session 1. *Excludes participants with a single time point (Wolfram, n = 4; Non-diabetic control = 6; diabetic control = 6). Abbreviations: M, male; F, female; dx, diagnosis.

#### Control group

**Figure 1:**
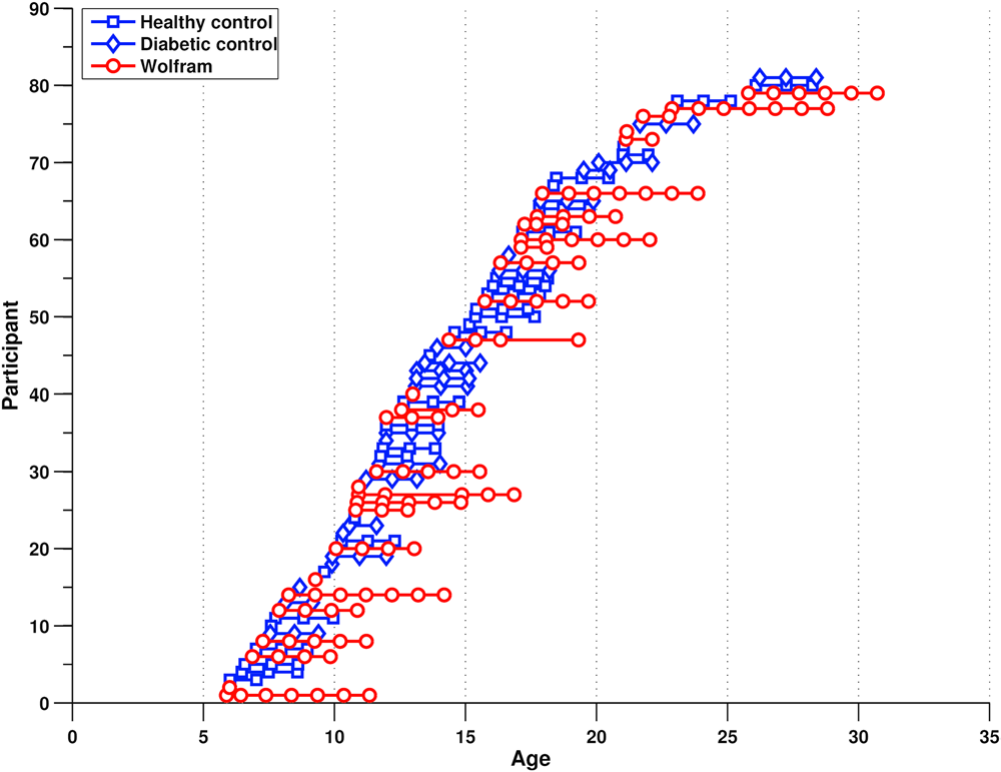
Ages of the 81 participants at each of their MRI scans. Each row on the Y-axis indicates an individual participant and symbols (connected by a line) indicate the participant’s age at each session (X-axis). Wolfram participants are shown in red and controls are shown in blue.

Adequate T1 and T2 images were acquired on 24 type 1 diabetics and 28 non-diabetic participants, annually between 1 and 3 times (mean follow-up = 2.0 years). The type 1 diabetes group had overall higher HbA1c than the Wolfram group (sessions 1–3, p < 0.022), however, these groups did not differ in blood glucose levels before or after scans (sessions 1–3, p > 0.23) or in diabetes duration (t(51) = −0.07, p = 0.95). Wolfram and control groups did not differ in age at their first visit (baseline age range 5–25 and 6–26, respectively; t(79) = 0.09, p = 0.93) or in sex distribution (χ^2^ (1, 81) = 0, p = 0.99). See Table 1 for demographics and Fig. 1 for a plot of all participant ages and number of sessions.

### Neuroimaging

#### Voxel-wise results

**Figure 2:**
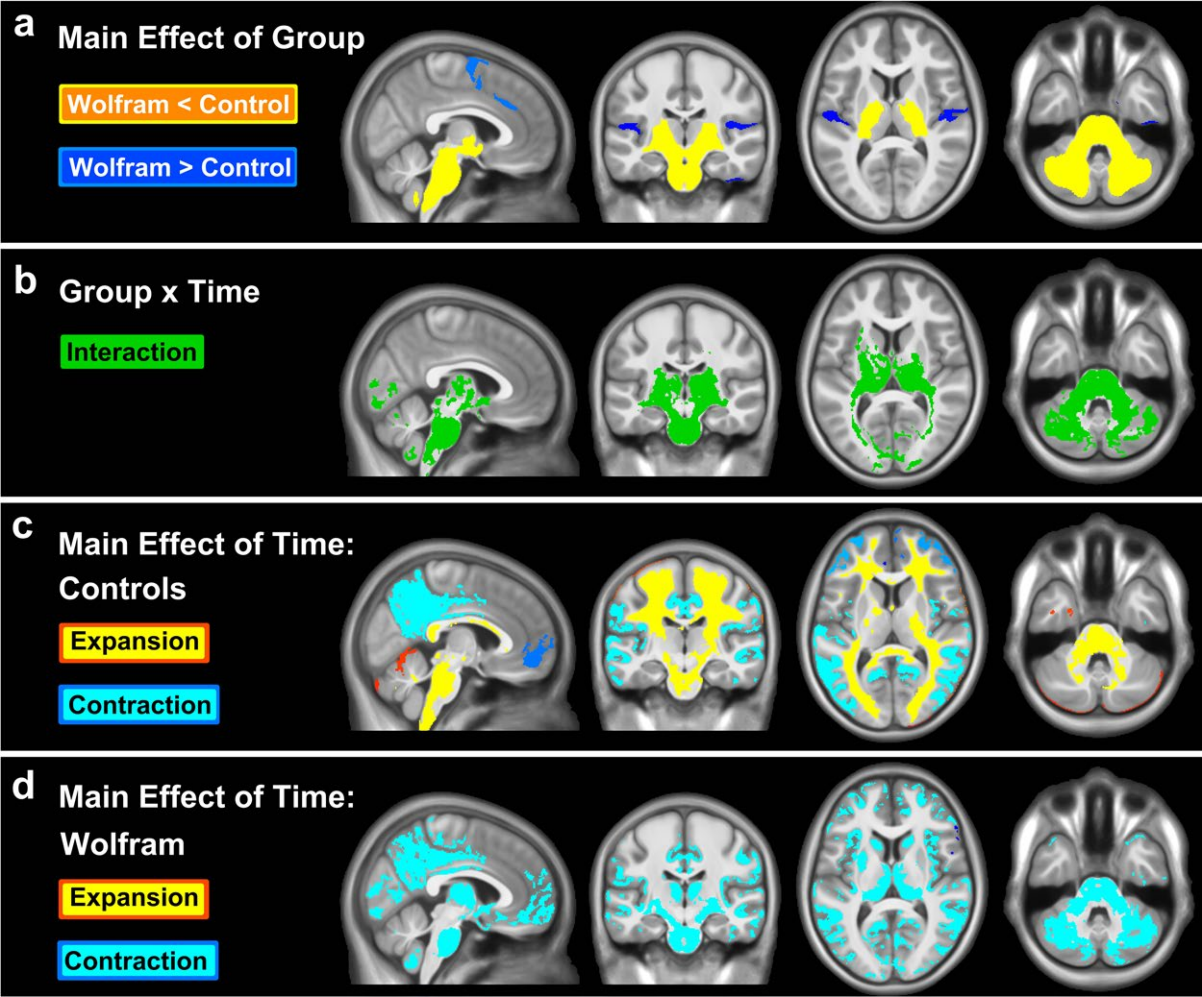
Clusters surviving FWE correction in each voxel-wise analysis at FWE p < 0.05. (**a**) *Main effect of group*: The Wolfram group has lower volume (yellow clusters) in multiple regions (e.g. brainstem, cerebellum, thalamus) and greater volume (blue) in relatively small portions of cortex compared to the control group. (**b**) *Group* × *time interaction*: Clusters where groups differed over time in volume (green) largely fell within the regions where the Wolfram group had lower volume than controls (yellow in **a**). (**c**) *Main effect of time within the control group*: Volume increased in most white matter regions (yellow and red clusters) and decreased in cortical gray matter regions (turquoise and blue) over time, as expected. (**d**) *Main effect of time within the Wolfram group*: Volume decreased over time (turquoise) in many white and gray matter regions, and no increased volume was seen anywhere in the brain. Expansion = increase in volume over time; Contraction = decrease in volume over time.

To confirm our previous cross-sectional neuroimaging findings in Wolfram syndrome, we first examined the main effect of group on cluster-wise bootstrap-corrected FWE P images. We found that the Wolfram group had lower volumes in the brainstem, cerebellum and thalamus and slightly higher volumes in restricted temporal lobe cortex compared to controls (Fig. 2a). These findings largely overlap with our previous cross-sectional results which used smaller sample sizes and different analytic methods^9,24^. The current analysis further revealed that the Wolfram group had lower volumes than controls in the cerebellar and cerebral peduncles and internal capsule.

No clusters from the group by time by age interaction survived cluster-wise FWE correction. Significant group by time interactions were found within the brainstem, notably in the ventral pons, posterior thalamus, internal capsule, optic radiations, a small portion of the visual cortex, most of cerebellar white matter, a portion of cerebellar gray matter, cerebellar peduncles, and cerebral peduncles (Fig. 2b). In order to better understand the nature of these interactions, we examined the voxel-wise main effect of time within each group separately. These maps indicate that within controls, as expected based on the literature, volumes increased across most major white matter regions, including brainstem and cerebellar white matter, whereas cortical gray matter volume decreased over time, particularly within the precuneus/cuneus and prefrontal cortex (Fig. 2c). In contrast, within the Wolfram group, white matter volumes did not show any increase over time. In addition, the ventral pons region of the brainstem and cerebellar white matter actually decreased in volume over time (Fig. 2d). Thalamus and cerebellar gray matter volume decreased in Wolfram over time, but was stable in controls.

#### Regions of interest (ROI) results

**Figure 3:**
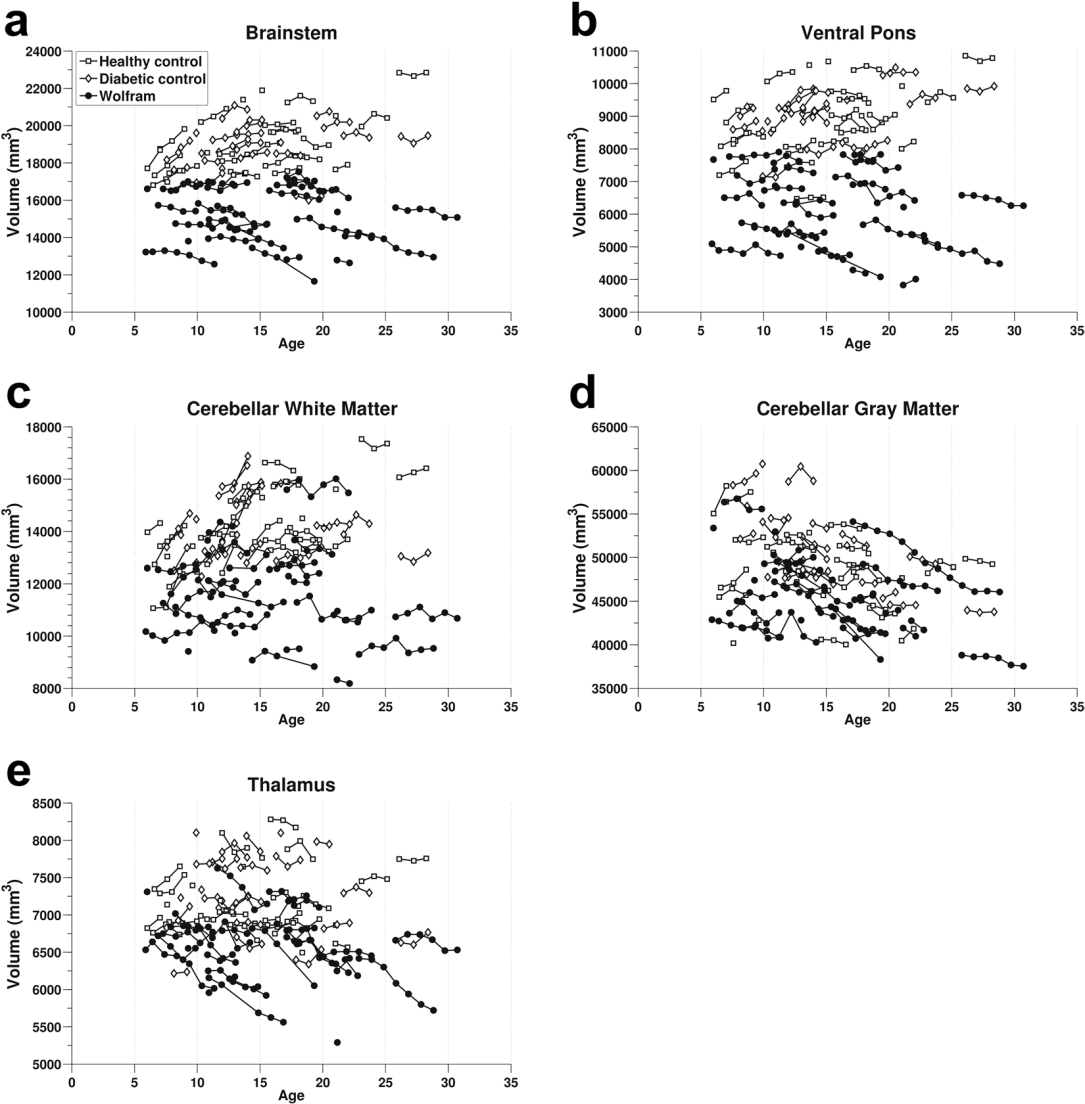
Volumes of regions of interests (corrected for eTIV) over time and age. All regions had a significant interaction of group by time. (**a**) Brainstem, (**b**) Ventral pons, (**c**) Cerebellar white matter, (**d**) Cerebellar gray matter, and (**e**) Thalamus. Freesurfer-derived volume is on the Y-axis and age is on the X-axis. Markers connected by a line denote age at each session for an individual over time. Open markers represent controls while solid markers represent Wolfram participants.

Based on the voxel-wise group by time findings, we selected brainstem, ventral pons, thalamus, cerebellar gray and cerebellar white matter regions. As expected, these regions all had significant interactions between group and time (p < 0.01; Table 2) and none had any group by time by age interactions, recapitulating the patterns observed in the voxel-wise analyses. In brainstem, ventral pons and cerebellar white matter, controls tend to increase in volume over time and age and the Wolfram group tend to decrease in volume over time and age (Fig. 3a–c). In the thalamus, we observed a striking decrease in volume in the Wolfram group over time while controls remained stable (Fig. 3e). Cerebellar gray matter decreased in both groups, but was more accentuated in the Wolfram group (Fig. 3d).

**Table 2:** Mixed model results for regions of interest.

Brain Region	Wolfram (n = 29)		Controls (n = 52)		Group × Time Interaction		Main Effect of Time: Wolfram	
	Mean	±SEM	Mean	±SEM	F	p	F	p
Brainstem	−123.61	26.08	182.39	17.71	70.03	**<0**.**001**	8.71	**0**.**006**
Ventral Pons	−48.44	18.33	93.23	12.34	31.69	**<0**.**001**	2.62	0.117
Cerebellar White	24.32	26.24	248.91	18.02	32.52	**<0**.**001**	1.49	0.232
Cerebellar Gray	−420.95	82.49	−49.26	60.43	6.83	**0**.**01**	13.42	**0**.**001**
Thalamus	−84.68	5.86	11.26	4.81	34.60	**<0**.**001**	49.47	**<0**.**001**

Mean and standard error of the mean (SEM) of the estimated annual rates of change are shown for each region and group. F and p values for each region come from the interaction of group by time, controlling for age, sex, and diabetes, and the main effect of time within the Wolfram group, controlling for age and sex. Significant results at the p ≤ 0.01 level are in bold.

**Figure 4:**
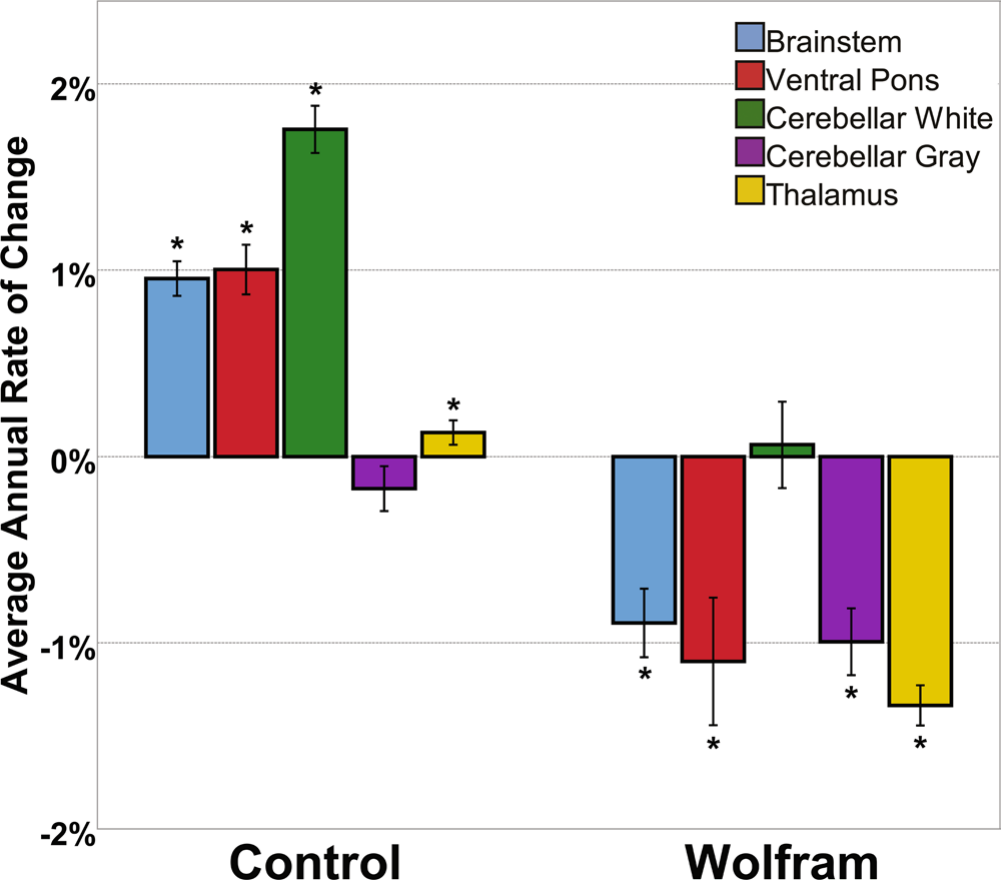
Mean (±SEM) estimated annual percent rate of change for regions of interest. Each individual’s estimated slopes from the mixed model analysis were scaled by their average volumes to calculate annual percent change for each region. Mean annual percent change for each region across groups is shown with the control group on the left and the Wolfram group on the right. Error bars represent standard error of the mean. *p < 0.05, one tailed t-test vs. 0.

**Figure 5:**
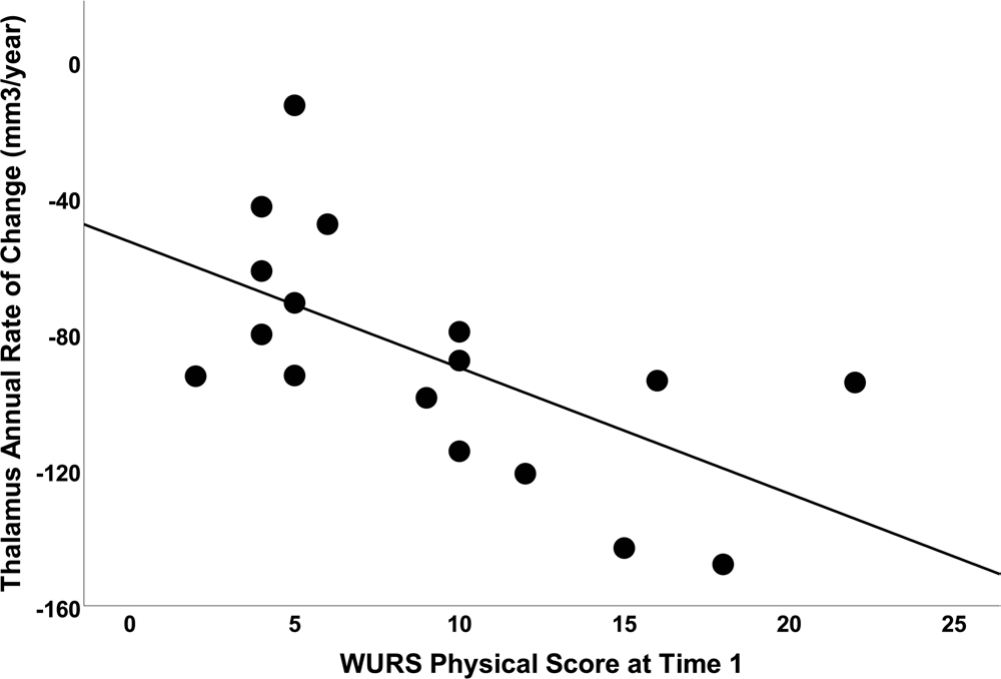
Correlation between percent change in thalamic volume and WURS physical score in Wolfram participants. Greater physical symptom severity (higher WURS score) at enrollment was associated with greater negative change in thalamic volume over time (r_s_ = −0.669, p = 0.003, n = 17).

Based on random slope models, the rate of change in each brain region was calculated for each person. As expected based on the group by time interactions from the mixed models, the average rate of change was significantly different between the two groups for all regions (p < 0.001). In addition, within the control group, average rate of change was different from 0 (p < 0.03) for each region except for cerebellar gray matter. Within the Wolfram group, the average rate of change was different from 0 (p < 0.02) for all regions except for cerebellar white matter (Fig. 4). To explore how degree of symptom severity at entry to the study predicted change over time, we performed Spearman’s correlations between annual rate of change variables and the WURS Physical Scale Score (n = 5 correlations). The correlation between thalamic rate of change and WURS Physical survived Bonferroni correction (criterion p value 0.05/5 = 0.01; r_s_ = −0.669, p = 0.003, n = 17; Fig. 5).

Within group mixed model analyses for the main effect of time within the Wolfram group determined that the largest effect was in the thalamus (F_1,28_ = 49.47, p < 0.0001; Table 2). Power analyses on the estimated annual rate of change in thalamus estimated that it would require 34 or 48 patients per study arm (treatment vs placebo) to detect a 60% or 50% slowing of the rate of volume loss due to an intervention.

## Discussion

Our longitudinal morphometric neuroimaging study of Wolfram syndrome identified the brain regions and tissue types that follow abnormal trajectories across development in this disease. Wolfram syndrome appears to be associated with deficient and stalled white matter growth and abnormal loss of white and gray matter in specific regions over time. The patterns observed suggest that there may be different mechanisms underlying the brain abnormalities present early in disease (hypo- and stalled myelination) vs. the evolution of abnormalities across development (axonal and neuronal degeneration). In addition, abnormal trajectories were found preferentially in the brainstem, cerebellum, thalamus and the white matter tracts that connect them while most of higher order cortex followed normal developmental trends in Wolfram syndrome. Finally, our findings suggest that thalamic volume is a novel and potentially useful biomarker for neurodegeneration in Wolfram syndrome, with the statistical power appropriate for small clinical trials. Together, these results suggest regionally specific neurodevelopmental and neurodegenerative processes at work that ultimately may explain the trajectory of neurological symptoms and provide data highly relevant to ongoing and future clinical trials in Wolfram syndrome.

Normal neurodevelopment, as reflected by tissue volume, is dynamic and multifactorial. In general, from childhood to early adulthood, white matter volume increases linearly, likely due to ongoing myelination of axons. In contrast, cortical gray matter volume peaks at an early age and then decreases over development, likely due to synapse elimination and dendritic pruning^41–44^. These processes also differ regionally, with higher order cortical regions developing later than primary sensory areas^43–46^. For example, primary visual and motor cortex reaches peak thickness around age 8, whereas prefrontal cortical gray matter reaches peak thickness around age 11, after which they decline to adulthood in a fairly linear manner^47^. Volumes for subcortical regions peak at about 13–19 years of age^48^ but the rate of increases and decreases are much more muted than for cortical gray matter^44,49^. Volumetric results from our control group recapitulated these known patterns in this age range, demonstrating mostly increasing volume in white matter, decreasing volume in cortical gray matter and relatively stable volume in subcortical gray matter. The complexities of these normal trajectories are an important backdrop for understanding the specific impact of Wolfram syndrome on the brain.

Over time, brain volumes in Wolfram syndrome appear to deviate from normal trajectories in a region and tissue-specific manner. Interestingly, we found no significant interactions with age, suggesting that group differences in trajectories are not dependent on the age of the individual. This result could mean that other factors are more powerful in driving individual differences in neurodegenerative processes in Wolfram than just age (e.g. severity of mutation) or that our sample is not large or diverse enough in age to be able to detect a 3-way interaction.

In white matter regions that increase in volume across normal development, the Wolfram group had stable (optic radiations, frontal white matter) or decreasing volume (cerebellar white matter, brainstem, pons). These findings suggest that Wolfram syndrome may interfere with the ability to myelinate existing axons over age and, in some regions, cause damage to myelin and/or axons. In addition, we observed lower white matter volumes at very early ages (e.g. 5), which may suggest an early lack of myelin and/or axonal development. Our previous cross-sectional diffusion tensor imaging (DTI) analyses were consistent with hypomyelination^24^, but longitudinal DTI analysis could help determine the impact of Wolfram syndrome on myelin vs. axons over time. It is possible that, as in other demyelinating diseases (e.g. multiple sclerosis^50^), lack of myelination precedes axonal damage, axonal loss and finally neuronal loss^28^. Longitudinal DTI analyses that may allow for these more detailed neurobiological interpretations are underway but outside the scope of this paper.

In gray matter regions that decrease in volume over this age range (cortex, cerebellum), the Wolfram group demonstrated a relatively normal trajectory of decreasing volume (cortex), except for a very small part of visual cortex and cerebellar cortex, which appeared to have accelerated decreases in volume. In normal development, decreased gray matter volume is thought to reflect synaptic and dendritic pruning^51^. Thus, it may be that Wolfram syndrome is associated with accelerated or excessive pruning or cell death in these restricted areas. In gray matter that is relatively stable over this age range (subcortex)^46^, the Wolfram group demonstrated an abnormal decrease in thalamic volume, but no differences in the other regions. We hypothesize that this pattern is most consistent with neuronal loss, perhaps due to loss or dysfunction of axons that project to or from thalamic nuclei as seen in other neurodegenerative diseases^52^. Interestingly, the voxel-wise findings appeared predominantly within the posterior thalami, which is composed of nuclei with inputs and outputs to the visual system, including pulvinar, lateral posterior and lateral geniculate nuclei^53^. Segmentation of thalamic nuclei might help us understand structure-function relationships and improve its use as a biomarker of neurodegeneration^54^. Further, rate of change in thalamic volume was predicted by disease severity (WURS Physical score) at time 1, suggesting that thalamic neuronal loss may be a later appearing sign in Wolfram syndrome, and have some clinical relevance. We determined that to detect a 50% slowing of thalamic volume loss, a clinical trial would require 34 patients per arm (treatment vs placebo) to achieve power of >0.80, which would be feasible even for an ultra-rare disease such as Wolfram syndrome.

Neuropathological studies of individuals with Wolfram syndrome are rare and findings have been somewhat variable. However, if these cases are arranged according to the age and severity of the patient, there does appear to be some support for an evolution of neuropathological findings from restricted myelin and axonal loss to widespread myelin, axonal and neuronal loss. Neuropathological examination of a 15 year old who died accidentally revealed reduced myelin and axons in restricted areas and neuronal loss only in the hypothalamus^11^. Exam results from a 21 year old with more extensive symptoms were similar but also included myelin and axon loss in the optic radiations and diffuse demyelination throughout the rest of the brain^11^. A 24 year old with advanced neurological symptoms at the time of death had widespread axonal loss and neuronal death^10^. Similarly, a 36 year old with severe neurologic symptoms at time of death had severe loss of myelin and presence of gliosis in the optic nerves and loss of myelin and neurons in the cerebellum^55^. Finally, a 38 year old patient with advanced symptoms at the time of death had axonal swelling and dystrophy, neuronal loss and gliosis in brainstem, cerebellum and optic nerves, and also neuronal loss and gliosis in thalamus^56^. Of note, these cases were all published before the age of genetic confirmation for Wolfram syndrome, used different methods and in some cases did not examine the entire brain, and so need to be viewed with some caution. Despite these caveats, it appears that cases with greater symptom severity may have more widespread axonal and neuronal pathology.

The functional consequences of these neurodevelopmental and neurodegenerative changes in Wolfram syndrome are unclear. However, we can speculate that white matter loss in the brainstem and pons could affect a wide range of motor, sensory and other essential functions (e.g. respiration, urination) known to be affected in Wolfram syndrome. In fact, in a cross-sectional, correlational analysis, we found that ventral pons volume correlated with the degree of urinary dysfunction^30^. In addition, gait and balance, which are associated with the cerebellum, are known to be abnormal in Wolfram syndrome, even at the early stages of the disease process^7,8^. Ultimately, we hope to understand whether change in neurological symptoms over time can be predicted by regional brain morphometry. Such information could strengthen the clinical relevance of these measures as outcomes for clinical trials, and lead to a better understanding of the key neuropathological changes in Wolfram syndrome that deserve further attention.

The major strength of this study is the longitudinal quantification of structural brain abnormalities in Wolfram syndrome compared to well-matched controls using voxel-wise and regional approaches. However, there are some limitations to our study. First, although all Wolfram group members who were willing to travel to St. Louis were accepted into the study, this requirement may have selected for individuals who were less severely affected than the general Wolfram syndrome population. Second, the maximum number of years that the control group was followed was shorter (n = 3) than for the Wolfram group (n = 7). Fortunately, mixed model statistical approaches are designed to handle this difference. In addition our primary results were quite robust and patterns can be seen clearly at the raw data level. Finally, the scope of this paper included longitudinal volumetric change in Wolfram syndrome. Future analyses will need to address longitudinal change in diffusion weighted imaging and behavior. We ultimately hope to determine whether rates of change in specific symptoms correlates with rate of change in specific brain regions or tissue types.

Our unique longitudinal neuroimaging dataset in Wolfram syndrome clearly indicate that while some morphometric brain abnormalities are present very early in the disease course, others appear to increase over time. The patterns observed suggest that both altered neurodevelopmental and degenerative processes occur in Wolfram syndrome distributed across region and tissue type. Animal models of Wolfram syndrome are improving^57^, with recent papers suggesting gross neurologic alterations that resemble the basic cross-sectional differences seen in human neuroimaging. The challenge remains to verify in animal models the longitudinal patterns seen here in our analyses and to determine whether there is a unifying primary mechanism that can explain both the spatial and tissue-specific patterns. Furthermore, understanding the predictive value of gene mutation for rate of progression of neurologic symptoms and brain degeneration is a future goal requiring larger samples. These issues have been explored in the past using retrospectively acquired basic clinical information (e.g. reported age of onset of symptoms)^6,58^. Future work will need to examine genotype-phenotype relationships using objective, quantified and longitudinal neuroimaging metrics, as presented in this paper, with a larger and more diverse sample.

## Data Availability

The raw data used in the analyses described in this manuscript cannot be made available in the manuscript, supplemental files, or a public repository because the sample size of our Wolfram syndrome patient group is so small, and the disease so rare, that human participant characteristics such as sex, age, and number of visits could be used to identify individuals even after de-identification of the data. The corresponding author, Dr. Hershey, may be contacted to request data. As per the Human Research Protection Office (HRPO) at Washington University, a preface to data sharing agreement and a data sharing agreement reviewed by the research office will be employed prior to data sharing. HRPO regulations permit access to potentially identifiable data only to research personnel on our study protocol and approved through the University.
